# Advances in Tissue Culture-Free Genetic Engineering and Genome Editing of Peanut

**DOI:** 10.1007/s12033-025-01476-8

**Published:** 2025-07-17

**Authors:** Tariq Alam

**Affiliations:** Department of Plant and Environmental Sciences, Clemson University Pee Dee Research and Education Center, Florence, SC 29506 USA

**Keywords:** Tissue culture-free methods, High-throughput transformation, CRISPR/Cas9 genome editing, Pollen, Magnetofection

## Abstract

Plant transformation and genome editing are pivotal in advancing peanut biotechnology, yet traditional tissue culture–dependent methods are hindered by lengthy protocols, genotype dependency, and somaclonal variation. CRISPR/Cas technologies have revolutionized breeding by enabling precise, multiplex genome editing to improve traits such as disease resistance, allergen reduction, seed quality, and yield. However, variable transformation efficiencies and chimerism remain challenges. This review examines emerging tissue culture–independent techniques such as nanoparticle-based delivery, viral vectors, pollen magnetofection, pollen tube injection, node injection, and vacuum infiltration that offer rapid, cost-effective gene transfer. It also highlights the integration of high-throughput screening, robust selection markers, and automation, including robotics and advanced imaging, to refine transformation pipelines. These methodological breakthroughs promise to overcome current limitations and accelerate the development of improved peanut cultivars for sustainable agriculture.

## Introduction

**Fig. 1:**
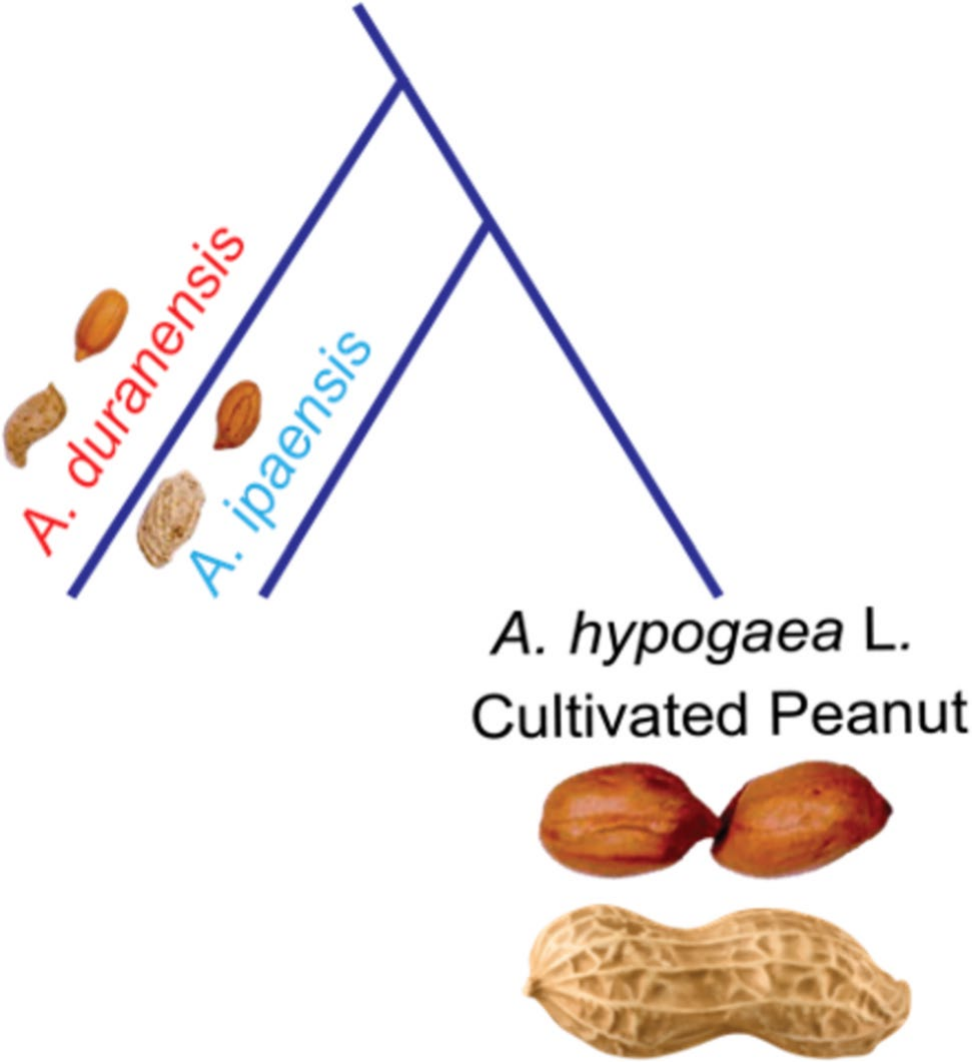
The origin of *Arachis hypogaea* L., the cultivated peanut, is attributed to the hybridization of *A. duranensis* and *A. ipaensis*, with their AA and BB genomes combining to produce a new tetraploid hybrid species

Peanut (*Arachis hypogaea* L*.*) belongs to genus *Arachis*. It has a complex allotetraploid genome comprised of 40 chromosomes (2*n* = 4x = 40). This species has a long cultivation history, spanning over thousands of years [64]. Peanuts are grown in tropical and sub-tropical agroecological regions of the world [37, 40]. The tetraploid cultivated peanuts are hypothesized to have originated from the hybridization between *A. duranensis* (AA genome) and *A. ipaensis* (BB genome), followed by an autonomous chromosomal duplication (Fig. 1) [38]. This process has resulted in the genomic structure observed in the modern peanut with A and B sub-genomes providing almost equal contributions to its DNA content [65, 82]. The detailed study of the genomic sequences suggested a single origin for cultivated peanut and explained the lack of diversity in cultivated peanuts and also the inexistence of many favorable alleles of genes conferring resistance to various biotic and abiotic stresses [14]. Genetically, the peanut exhibits diploid behavior despite its allotetraploid nature [67]. The tetraploid peanut genome is approximately 2.7 gigabases (Gb), where A sub-genome (1.2 Gb) comprise 36,734 genes and B subgenome (1.5 Gb) 41,840 genes [84]. Most species of wild *Arachis*, such as *A. diogoi, A. batizocoi*, and *A. cardenasii,* possess a diploid chromosome count (2*n* = 20) [67]. *Arachis* species hail from South America, spanning the territories of Argentina, Brazil, Bolivia, Paraguay, and Uruguay [71].

Peanut is an annual herbaceous allotetraploid crop that stands 30–50 centimeters (cm) tall. Its distinctive pinnate leaves, arranged in opposite pairs, consist of four leaflets but lack a terminal leaflet [30, 39, 56]. The foliar parts of the plant exhibit nyctinastic movements, closing during the nocturnal hours [10, 70]. The reproductive process of the peanut plant is characterized by geocarpy, where the small yellow flowers are produced above the ground, leading to fruit development beneath the soil. The gynophore, or peg, which is the main reproductive organ, emerges from the pollinated flowers and pushes the fertilized ovules into the soil, typically within one week after pollination [13]. The gynophore penetrates the soil to a depth of 2–8 cm, reaching an area referred to as the pegging zone, where the embryo enclosed begins the nutrient absorption process. This results in the enlargement of embryo and subsequent seed formation within the confines of the protective pod [13, 54, 58]. During its development, the peanut pod undergoes a phase of active enlargement, during which it assimilates nutrients, including calcium and various inorganic salts, along with moisture [13, 29, 63]. Peanuts exhibit optimal growth in soil with a pH range of 6.2–6.5 [1, 80]. Typically, the pod of the peanut plant contains between one and four seeds. Peanut cultivars are classified into four principal market categories: Runner, Virginia, Spanish, and Valencia [69]. This classification is based on phenotypic traits such as seed size, taste, and utility. The Runner variety, which dominates U.S. peanut production, is selected for processing into peanut butter owing to its optimal kernel size. The Virginia type is characterized by its large seeds, making it preferred for roasting and direct consumption. Spanish peanuts, noted for their smaller seeds and high oil content, are typically utilized in confectionery and oil extraction industries. Valencia peanuts are distinguished by their sweet flavor and the presence of multiple small seeds per pod, making them suitable for crafting artisanal peanut butter and for culinary uses such as boiling (American Peanut Council [7]).

## A Brief History of Peanut Transformation

It has been over four decades since the pioneering transgenic plant was first developed, and in the years since, nearly every commercially important crop has been transformed at least once, leading to the widespread adoption of transgenic hybrids and cultivars in species such as corn, canola, soybean, and cotton [36, 62]. Plant transformation is a vital tool in functional genetic research, enabling the identification of biological processes and traits that biotechnology can leverage to advance crop genetic improvement [6]. Over the past years, plant genetic transformation has advanced significantly. However, a major challenge remains in developing complementary tissue culture methods, which are essential for successful transformation. The refinement of tissue culture techniques is a complex process that requires optimization at multiple stages, including the isolation of cells or specialized tissues, their controlled growth under aseptic conditions, the establishment of transformation protocols using *Agrobacterium spp.* or alternative methods such as biolistic delivery, polyethylene glycol-mediated transformation, or electroporation, and ultimately, the regeneration of transformed plants. Due to the intricate nature of these processes, genetic transformation and plant regeneration via tissue culture are both time-intensive and labor-intensive. For instance, even with well-established protocols, peanut transformation can take approximately 14–16 months to complete [4]. A potential limitation of tissue culture-based approaches is somaclonal variation, which introduces unintended genetic modifications that may be undesirable for researchers requiring genetically stable clones [8].

**Fig. 2:**
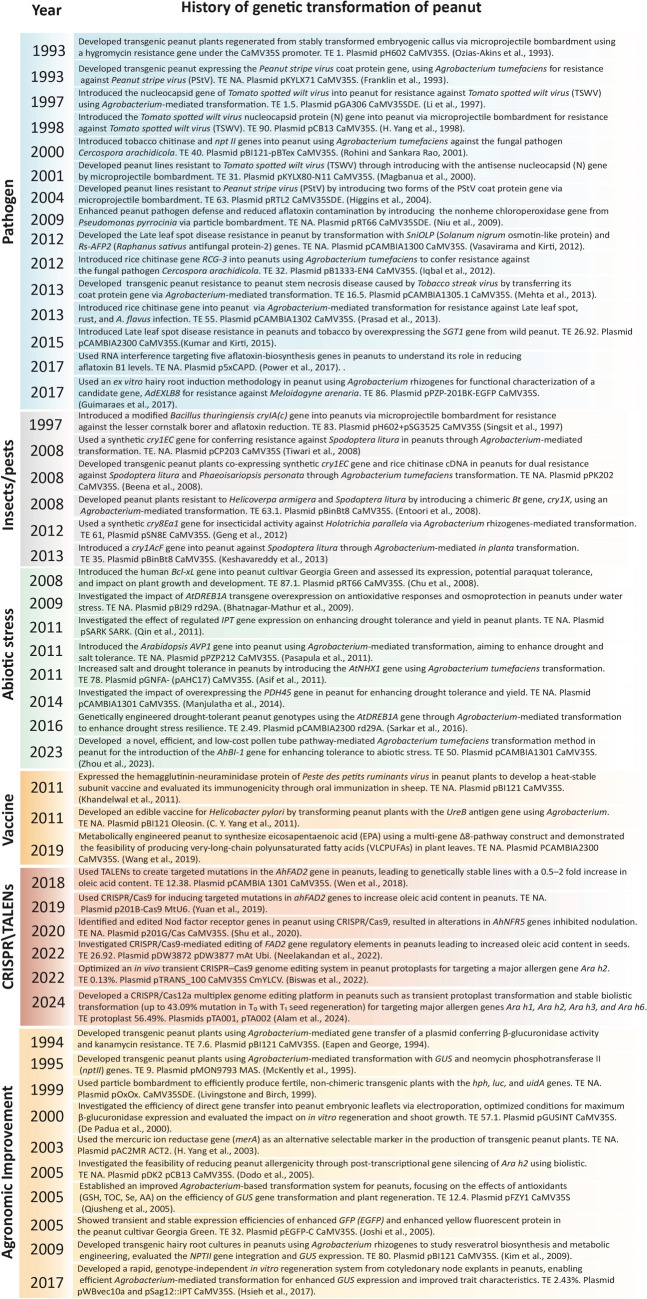
Peanut transformants developed for different traits between 1993 and 2024. Note: TE denotes transformation efficacy; NA indicates data not available; plasmid name is followed by promoter

Globally, peanut yield and productivity are significantly impacted by biotic stressors, such as pathogens and insect pests, and abiotic factors, including drought, salinity, waterlogging, and temperature fluctuations. Genetic engineering techniques, such as *Agrobacterium*-mediated transformation and biolistic DNA delivery, play a crucial role in complementing traditional breeding methods to improve peanuts by integrating desirable agronomic traits into high-yielding varieties. Successful efforts have been made to confer resistance to a variety of fungal, viral pathogens and insect pests over the years. Figure 2 provides a historical overview of peanut genetic transformation efforts from 1993 to 2024.

Since the early 1990 s, beginning with Ozias-Akins et al.'s [52] demonstration of peanut transformation via particle bombardment and Franklin et al.'s [21] pioneering introduction of the *Peanut stripe virus* coat protein gene through *Agrobacterium*-mediated methods, peanut genetic engineering has experienced significant growth. Milestones include Singsit et al.'s [66] innovative work on pest resistance and aflatoxin reduction, and more recent advancements in genome editing and metabolic engineering by Yuan et al. [83], Wang et al. [75], and Alam et al. [3] demonstrated that CRISPR/Cas12a multiplex gene editing can effectively induce mutations in the major peanut allergen genes, offering a promising approach to develop peanut genotypes with reduced immunogenicity.

## Peanut Gene Delivery Methods: Transitioning from Traditional to Next-Generation

Gene-delivery methods include classical approaches, *Agrobacterium*-mediated transformation, particle bombardment, and next-generation technologies. Next-generation gene-delivery technologies comprise (i) nanomaterial-enabled transport of nucleic acids or CRISPR ribonucleoproteins (RNPs) across the peanut cell wall; (ii) engineered plant viruses or virus-like particles that transiently express editing reagents without genomic integration; and (iii) developmental-regulator or *in planta* approaches that induce de novo meristems, thereby bypassing the callus phase. To successfully produce transgenic plants, two critical steps must be executed precisely: transformation, the introduction and expression of foreign genetic material in host cells, and regeneration, where these transformed cells develop into fully fertile plants. In many plant species, the main challenges in generating transgenic lines stem from these critical stages. Traditionally, callus induction from an explant has been the cornerstone of transgenic plant development, as it provides a reservoir of undifferentiated cells amenable to genetic transformation and supports the regeneration of complete plants from a limited number of transformed cells.

**Fig. 3:**
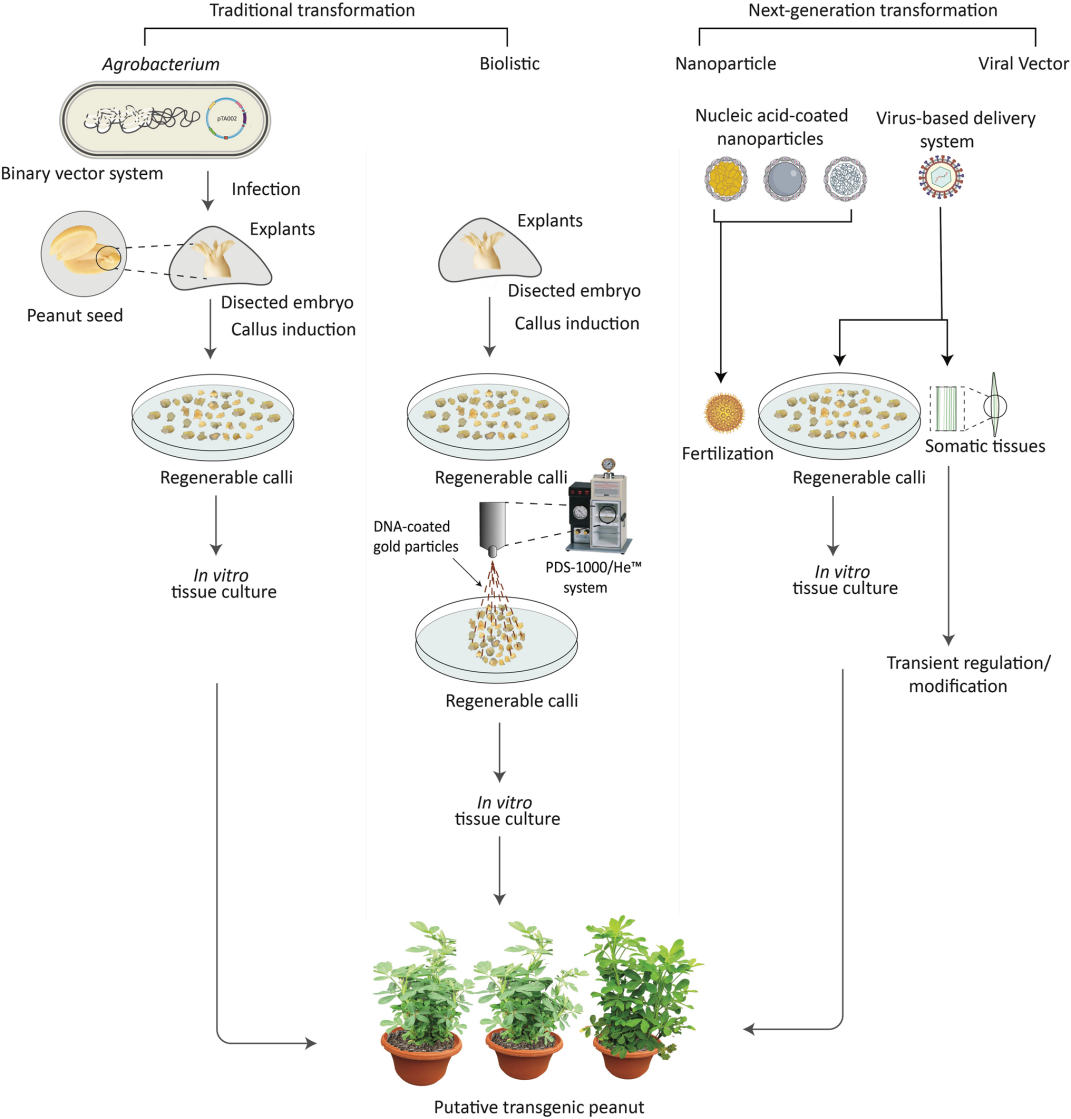
Diagrammatic representation of different methods used for plant genetic transformation. Left, *Agrobacterium*-mediated transformation involves co-culturing embryogenic calli with *Agrobacterium* for plant genetic transformation. Middle, biolistic delivery involves bombardment of embryogenic calli with DNA-coated gold particles. Right, nanoparticle and viral delivery systems are utilized for *in planta* delivery of DNA for genetic transformation

A pivotal initial step in plant transformation and genome editing is the effective introduction of gene-editing reagents into plant cells. Since the 1980 s, two primary methodologies for gene delivery in higher plants have been established: direct gene transfer (biolistic approach) and *Agrobacterium*-mediated transformation [61]. Direct gene transfer methods serve as effective strategies to bypass potential genetic compatibility barriers in transformation. Traditionally, microprojectile bombardment, commonly referred to as biolistics, has been employed for transgene delivery across a wide range of species. Plants derived from particle bombardment often exhibit multiple integration events and random rearrangements of the integrated genetic copies. These phenomena can lead to unpredictable effects, thereby complicating the subsequent analysis of the resulting transgenic plants (Fig. 3) [44]. Nonetheless, these events are not solely associated with biolistic methods. Such effects have also been reported in cases of *Agrobacterium*-mediated transformation, but to a limited extent [16, 26, 31, 41, 55]. In recent developments, the biolistic method has been adapted to deliver in vitro-created transcripts or CRISPR-Cas9 ribonucleoprotein complexes directly into regenerable plant tissues. This technique is now applicable to a broad range of genotypes and species, including *Arabidopsis thaliana*, tobacco, lettuce, rice, wheat, and maize, bypassing the effects associated with genome integration [42, 43, 68, 79].

*Agrobacterium*-mediated transformation is a cost-effective and efficient approach for gene delivery, known for its ability to transfer large DNA fragments into plant chromosomes, making it the preferred choice for plant transformation. However, its effectiveness is contingent upon the host range of *Agrobacterium* strains used for gene delivery. For instance, in many monocot crops *Agrobacterium*-based gene delivery has been found to be less effective [24]. Nevertheless, recent advancements have led to modified *Agrobacterium* strains equipped with a type III secretion system capable of delivering *Pseudomonas* effectors. These modifications have shown promising results, enhancing transformation efficiency in wheat, alfalfa, and switchgrass by inhibiting host defense responses [57].

Viral vector systems offer a significant advantage by circumventing transgene integration in the host plant genome. This allows quick characterization of target genes and bypasses regulatory hurdles. These systems have been modified to facilitate genome editing across various species by efficiently delivering and expressing guide RNAs (Fig. 3). DNA viruses such as geminiviruses (e.g., BeYDV, CaLCuV, TYLCV) can be converted into replicons that transiently amplify both Cas nucleases and donor templates; this strategy boosted homology-directed repair three-fold in tomato and yielded transgene-free, precisely edited progeny [74]. Among RNA viruses, negative-strand rhabdoviruses such as *sonchus yellow net virus* possess a large cargo capacity and have been engineered to carry the complete Cas9 and sgRNA cassette, producing heritable edits in *Nicotiana benthamiana* within a single generation without any tissue culture step [49]. Most recently, a *tobacco rattle virus* (TRV) delivery platform was engineered to carry the ultra-compact ISYmu1/TnpB nuclease. A single agro-infiltration produced transgene-free, heritable edits in *Arabidopsis*, demonstrating that reducing editor size can overcome viral cargo limits [78]. Viral vectors such as *bean-pod mottle virus* and *apple latent spherical virus* already deliver guide RNAs in soybean, demonstrating cross-family portability that is directly applicable to peanut improvement efforts [11, 47]. Combined with our engineered *tomato yellow leaf curl virus* (TYLCV) replicon vacuum infiltration system delivering CRISPR/Cas12a (pTA001) into peanut plants, achieving transformation efficiencies of ~ 70% for *Ara h1*, 100% for *Ara h2*, 90–100% for *Ara h3*, and 10% for *Ara h6*, these advances position viral vectors as a powerful, high-throughput gateway to genotype-independent peanut genome editing (Alam et al. 2024). Despite limitations, such as host range and inability to infiltrate the reproductive tissues, these virus-based systems hold great promise for genome editing of agricultural crops.

Over the past two decades, nanoparticles have been increasingly employed as vehicles for gene delivery across various genome editing (GE) methodologies [2, 19, 45]. Nanomaterials like silica, metal, polymer, magnetic nanoparticles, and carbon nanotubes are being studied for their role in gene delivery for plant genetic transformation (Fig. 3) [48]. Nonetheless, nearly every nanoparticle-mediated GE system continues to depend on tissue culture techniques for regenerating transgenic plants [2, 51, 59]. Recent advancements, however, are paving the way for tissue culture–free approaches. For instance, tissue culture–free techniques like pollen magnetofection enable direct gene transfer into viable pollen, potentially streamlining the development of transgenic lines and overcoming the inherent limitations of conventional protocols (Table 1).

**Table 1:** Recent advances in tissue culture-free transformation methods for genetic modification and genome editing in peanut

Method	Delivery target	Demonstrated species	Transformation efficiency	Time to regeneration/throughput	Advantage	Potential improvement	References
Vacuum infiltration	Roots	Peanut	~ 70% for *Ara h1*, 100% for *Ara h2*, 90–100% for *Ara h3*, and 10% for *Ara h6*		Exploits the plant vascular system for systemic delivery, Versatile	Viral vector delivery parameter, such as vacuum pressure and infiltration time	Alam et al. (2024)
Pollen-tube injection	Pollen Tubes	Peanut	2.6%	Transgenic seeds obtained from mature pods (~ 100-day-old plants)	Cost-effective & scalable	Increase efficiency, optimization of injection parameters, refine environmental control	Huang et al. [28]
*In planta*	Seed	Chickpea, Pigeon pea, Wheat, Indica rice, Soybean, Okra, Gladiolus, Cotton	14.3–40.9% in chickpea, 45.0% in pigeon pea, 27.0–58.9% in wheat, 93.8% in indica rice, 51.3–75.0% in soybean/okra/gladiolus, and 35% in cotton		Simple, cost-effective & rapid	Optimize culture conditions	Kharb et al. (2024)
Genotype-independent fast transformation (GiFT)	Seed	Soybean	2–30% (varies by genotype and construct)	~ 35 days from seed imbibition to fully established plants	Rapid regeneration, Cost-effective & high-throughput, Versatile	Further boosting efficiency, Standardization across genotypes	Zhong et al. [86]
*In planta*	Seed	Peanut	Ranges from ~ 31.3% (cv. CO7) to ~ 38.6% (cv. TMV 7);	~ 40 days	Cost-effective & high-throughput, Versatile	Further optimization of physical and chemical parameters to boost efficiency	Karthik et al. [33]
Node injection transformation	Nodes	Peanut	50%	~ 4–5 months	Simple, cost-effective & rapid	Optimize injection timing & sites	Han et al. [25]
Pollen tube transformation (PTT)	Pollen Tubes	Peanut	50%	Transgenic seedlings identified by the fourth true leaf and later at 27 days old	High Efficiency, Rapid Screening	Optimization of injection protocol, expansion to other cultivars	Zhou et al. [87]
Cut–dip–budding (CDB)	Stem, Root tissue	Russian dandelion, Sweet potato	Russian dandelion: ~ 40%; Sweet potato: ~ 27%	Russian dandelion: Hairy roots in ~ 2 weeks; bud formation ~ 1 week; sweet potato: 1–4 months	Cost-effective and rapid, Broad applicability	Apply an excision strategy to effectively remove the integrated oncogenes. Scale up for wider application	Cao et al. [15]
*In planta*	Shoot Apical Meristem	Peanut	~ 35%	Seedlings grown aseptically for 6–7 days	Direct integration into regenerable tissue	Scale up and refine screening, enhance selection protocols	Keshavareddy et al. [34]
Pollen Magnetofection	Pollen Grains	Cotton, Pepper, Pumpkin, Lily	2–12%	Less than 6 months	Preserved Pollen Viability, Rapid & Scalable	Refining timing and methods to enhance pollen adherence and fertilization efficiency, vector stability enhancements	Zhao et al. [85]
*In planta*	Seed	Peanut	3.3–4%	~ 4 weeks	Rapid recovery of transgenic plants, easy to apply across different peanut cultivars	Enhancing transformation efficiency, alternative selection markers to avoid growth inhibition	Rohini and Rao [60]

**Fig. 4:**
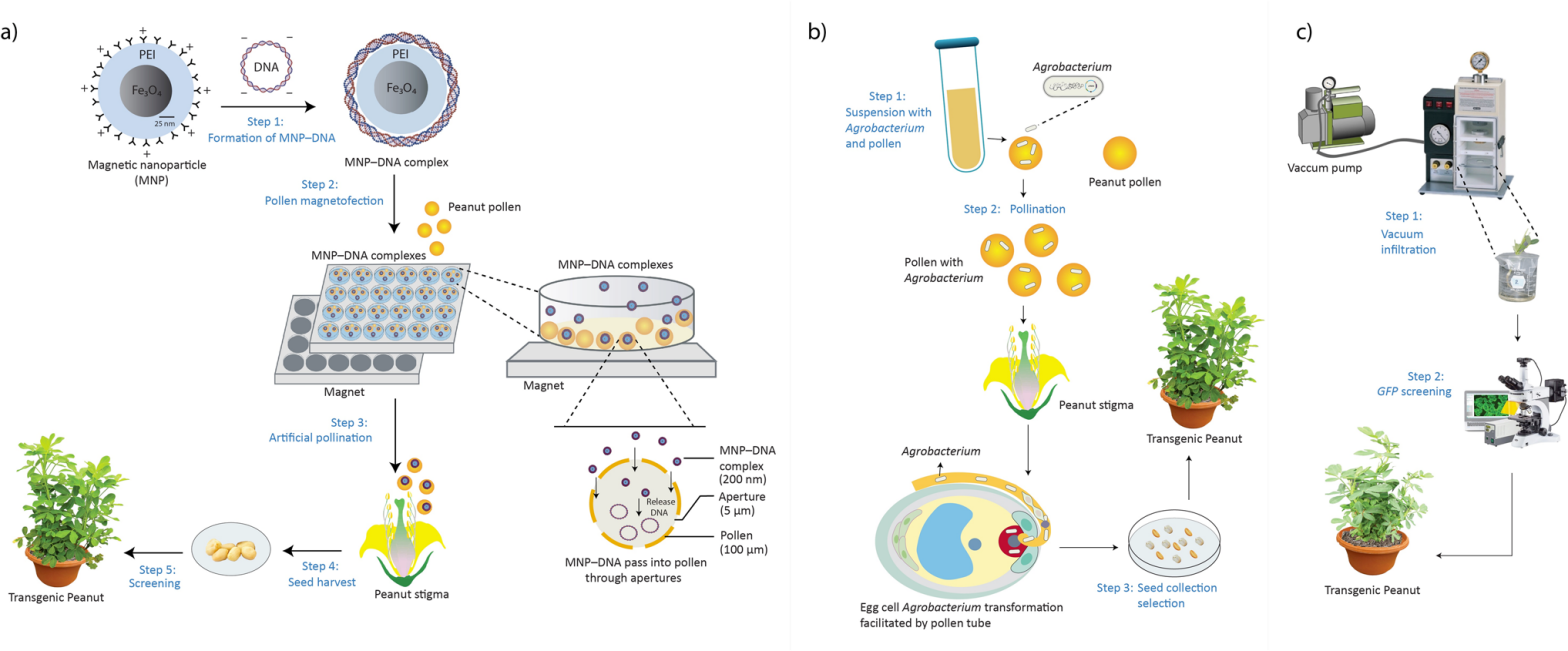
Schematic Overview of *In planta* Gene Delivery Techniques for Peanut Transformation. **a** Pollen magnetofection: plasmid DNA is electrostatically bound to magnetic nanoparticles (MNPs) and introduced into pollen grains under a magnetic field; fertilization with treated pollen produces transgenic seeds. **b** Pollination-based infiltration: pollen is exposed to *Agrobacterium* or exogenous DNA solutions before pollination, allowing gene transfer directly through the flower’s reproductive tissues. **c** Vacuum infiltration: a negative pressure is applied to draw the DNA or *Agrobacterium* suspension into plant tissues (e.g., roots or leaves), facilitating systemic movement of the transgene and subsequent screening of transgenic progeny

Pollen magnetofection refers to a recently established gene transfer system wherein plasmid DNA is first electrostatically attached to positively charged magnetic nanoparticles and subsequently directed into pollen via a magnetic field (Fig. 4a) [85]. Unlike conventional methods that rely on physical disruption [9] or bacterial infection [35], pollen magnetofection capitalizes on naturally occurring apertures in the pollen wall, through which magnetically guided DNA–nanoparticle complexes can enter. Because pollen is already primed for fertilization, the method enables the swift and straightforward generation of transgenic seeds once pollination is performed [85]. This innovative procedure provides a promising alternative for peanut transformation by bypassing tissue culture, making it particularly valuable for peanut cultivars that have been historically recalcitrant to traditional methods.

The success of pollen magnetofection depends on several critical factors. First, freshly collected pollen must be used to preserve its high viability and fertilization capability. Second, achieving the optimal DNA-to-nanoparticle ratio is vital; circular plasmids are generally favored for their enhanced stability against nuclease attack. Third, the process requires a well-calibrated magnetic field applied for an appropriate duration to drive the nanoparticles into the pollen grains effectively. Moreover, maintaining ideal environmental conditions, particularly controlled humidity and temperature, help sustain pollen health. Finally, careful pollination techniques are required to transfer an adequate amount of treated pollen to the stigma, thereby increasing the likelihood of generating transgenic progeny [85].

Originally developed in cotton, pollen magnetofection can be adapted for peanut by accounting for its unique flower biology and pollen physiology. The process involves collecting pollen at peak viability, typically early in the day, and formulating DNA–nanoparticle complexes. Fresh pollen is then exposed to a magnetic field for approximately 30 minutes before being promptly used for pollination, a critical step given peanuts'brief bloom period and rapid transition to peg formation. The mature pods are harvested, and the resulting seeds are screened for transgene integration.

However, recent studies indicate that the pollen magnetofection technique exhibits limited efficacy in certain monocot species, including sorghum, maize, and lily, even when the published protocol is strictly followed [73]. This reduced performance is primarily attributed to the maize pollen operculum, which covers the pollen aperture and thereby obstructs the entry of exogenous materials. As a result, pretreatment of pollen with a transfection buffer at 8 °C has been shown to modify the aperture and increase the number of accessible pores [77]. Similarly, ultrasonication has been employed to remove the operculum from maize pollen [81]. However, this method is associated with a decrease in seed set per ear, most likely due to diminished pollen viability [81]. Moreover, environmental factors such as humidity and temperature are hypothesized to influence the proportion of open apertures in pollen from field-grown versus greenhouse-grown plants, affecting aperture status at specific developmental stages. Therefore, evaluating pollen status using techniques like scanning electron microscopy (SEM) is critical before applying pollen magnetofection in monocot species with operculate pollen [22, 77]. Currently, researchers are investigating the application of pollen magnetofection for peanut genetic transformation [18]. Their study may reveal whether the obstacles observed in monocots are relevant to peanut pollen and help refine the technique for a rapid, tissue culture–free approach to peanut genetic transformation.

## Transformation with Germinating Seeds or Explants

The first successful tissue culture–free, *Agrobacterium*-mediated stable transformation was achieved by using imbibed and germinating seeds as transformation targets, a method first demonstrated by [20]. By bypassing extensive callus formation, this innovative approach enabled the direct introduction and expression of transgenes *in planta*, thereby significantly reducing labor and time requirements. This breakthrough laid a robust foundation for subsequent advances in plant genetic transformation technologies. Building on these foundational achievements, a GiFT (Genotype-independent fast transformation) *Fast* method was developed for soybean, representing a significant advancement in plant genetic transformation (Table 1). In this protocol, the seed coat is removed from imbibed seeds, and one cotyledon, along with the primary leaf primordia, is excised. The cotyledonary node region is then deliberately wounded, typically via brief sonication in an *Agrobacterium* suspension, to facilitate bacterial penetration into the meristematic tissues. Following inoculation, the explants undergo a short incubation in a liquid medium containing a sublethal concentration of herbicide, which serves as an initial selection pressure by inhibiting non-transformed cells. Subsequently, the explants are transferred to soil and maintained under continuous herbicide spraying for three weeks. This selective regimen promotes the outgrowth of transformed shoots while preserving the original root system. Overall, this method yields healthy, fully transgenic soybean plants in approximately 35 days, substantially reducing the dependence on labor-intensive tissue culture procedures and providing a genotype-flexible platform for plant transformation [86]. Hence, the GiFT method is a rapid and efficient approach for generating transgenic plants with minimal *in vitro* manipulation. Given that peanut exhibits a comparable cotyledonary node organogenic capacity, a GiFT-based protocol could be adapted for peanut transformation. This adaptation would involve optimizing several parameters, including seedling preparation, wounding techniques, and herbicide concentrations, to ensure effective *Agrobacterium*-mediated gene transfer. By leveraging the endogenous regeneration pathways of peanuts, such a protocol may eliminate the need for complex tissue culture procedures, thereby streamlining the production of transgenic peanut plants. In peanuts, a tissue culture-independent, *Agrobacterium*-mediated transformation method has been developed. In this protocol, a cotyledon is excised from mature seeds, and the exposed embryo axes are subsequently infected with *Agrobacterium* that has been supplemented with wounded tobacco leaf extract to enhance transformation efficiency. Transgenic plants are then identified through β-glucuronidase (GUS) assays and confirmed by PCR analysis. This approach achieved a transformation efficiency of approximately 3.3% and demonstrated stable gene inheritance across both T1 and T2 generations [60]. The reliance on direct organogenesis in this protocol demonstrates the feasibility of achieving stable transformation events without the complexities often associated with callus-based systems. Furthermore, a Temporary Immersion System (TIS) exemplifies another innovative strategy for improving plant regeneration. TIS is a liquid culture technique that intermittently immerses plant explants in a nutrient-rich medium, thereby optimizing nutrient absorption and gas exchange while mitigating hyperhydricity. In the context of peanut regeneration, TIS enabled efficient, genotype-independent organogenesis from de-embryonated cotyledon explants. The automation potential of TIS further streamlines the regeneration process, facilitating the scalable production of uniform plantlets. Regeneration efficiency, evaluated by the percentage of explants producing shoots and the degree of shoot proliferation, was significantly enhanced across four peanut cultivars (Virginia, NC7, 7 × 77, and Com74) compared with conventional semi-solid media. These findings highlight the efficacy of TIS in not only optimizing regeneration parameters but also advancing high-throughput applications in plant transformation [53].

## Young Seedling and Whole Plant Transformation

Whole-plant transformation strategies present a promising alternative to conventional plant genetic modification, addressing many limitations linked to tissue-culture-based protocols. In addition, these strategies reduced timeframes and increased scalability, thereby expanding the possibilities in plant genetic engineering, making them a powerful tool for genetic engineering. In a recent report by Han et al. [25], a genotype-independent node injection procedure was combined with CRISPR/Cas9 gene editing to modify the *FAD2B* gene in the peanut cultivar Huayu 23 (Table 1). This technique yielded a high-oleic phenotype, with oleic acid levels exceeding 80%, while effectively circumventing the limitations associated with traditional tissue culture methods. Heritable genetic modifications were verified via bar gene amplification, confirming stable transmission of the edited trait across subsequent generations [25]. Vacuum infiltration generates negative pressure, which extracts air from microcavities on the explant surface, thereby creating hollow spaces. This phenomenon facilitates the introduction of the genome-editing plasmid construct into these voids under controlled increased pressure, thereby ensuring efficient penetration and transformation of the meristematic cells within the explant (Fig. 4c) [50]. We demonstrated the effectiveness of this approach by delivering a TYLCV-based genome-editing vector (pTA001) into young peanut seedlings, thereby confirming its practical benefits over traditional methods [4]. In our protocol, leaves were trimmed, root tissue was sterilized, and the roots were subjected to reduced atmospheric pressure (150 mbar) for one minute to ensure robust uptake and systemic movement of the plasmid. Subsequently, confocal microscopy was performed to confirm *GFP* fluorescence in vascular tissues, PCR with GFP-specific primers amplified the transgene, verifying its integration, and PCR with TYLCV coat protein–specific primers amplified the viral coat protein gene in T₁ plants. Sanger sequencing confirmed targeted mutations in multiple peanut allergen genes (*Ara h1, Ara h2, Ara h3, and Ara h6*), with InDel frequencies ranging from 10 to 100%, thereby underscoring the efficiency of the infiltration [3]. Interestingly, a narrower range of small deletions (− 1 to − 6 bp) was observed *in planta* compared to protoplast-based transfection edits, suggesting potential avenues for fine-tuning the vacuum infiltration procedure [3]. Overall, these findings highlight the feasibility of vacuum infiltration for direct delivery of genome-editing reagents to peanut plants, offering a rapid and potentially scalable approach that could accelerate both functional genomics studies and the development of desired crop traits (e.g., reduced immunogenicity) without the need for traditional tissue-culture-based transformation systems.

Another *in planta* system utilizing *Agrobacterium* tumefaciens on half-seed explants demonstrated impressive transformation efficiencies (31.3–38.6%) across five peanut cultivars (CO7, CO6, TMV2, TMV7, and VR13) [33]. Similarly, Wang et al. [76] documented transformation frequencies ranging from 2.08 to 86.51% in four other cultivars (Huayu 22, Huayu 40, Huayu 20, and Fakusilihong) using a node injection strategy [75]. Furthermore, a recently reported genotype-independent method for developing transgenic lines in various crops, including chickpea, pigeon pea, wheat, rice, soybean, okra, and gladiolus. The method utilizes dry, mature seeds for transformation, eliminating the need for tissue culture. Transformation efficiency ranged from 16 to 93.8%, depending on the crop and *Agrobacterium* strain [32]. This approach demonstrates broad applicability across different genotypes, making it suitable for crops with tissue culture recalcitrance. Given its high efficiency and genotype independence, this protocol could be adapted for peanut transformation with optimization of parameters such as co-cultivation time, *Agrobacterium* strain, and acetosyringone concentration. In another notable report, [34] introduced an efficient *in planta* transformation strategy for peanut cv. K-134. In this protocol, two-day-old seedlings serve as explants, the shoot apical meristem is precisely targeted using a needle-pricking method and then immersed in an *Agrobacterium* suspension containing the chimeric *cry1AcF* gene. This method achieves a transformation efficiency of about 35%. Although the study was conducted solely on the K-134 cultivar, the methodology’s reliance on direct transformation of the apical meristem rather than on conventional tissue culture techniques, which are frequently constrained by genotype specificity, suggests that this approach could be broadly applicable across diverse groundnut genotypes. More recently, Cao et al. [15] introduced a novel cut–dip–budding (CDB) system that also circumvents the limitations imposed by tissue culture bottlenecks. In this approach, wound sites on seedlings, specifically, stem segments near the shoot–root junction in *Taraxacum kok-saghyz* and apical stem cuttings in sweet potato are directly inoculated with *Agrobacterium* rhizogenes (Table 1). Subsequently, the infected tissues develop transgenic hairy roots that spontaneously generate gene-edited buds and shoots via root suckering. When applied to six plant species from different families, this CDB method proved effective in sweet potato as well, successfully transforming ten cultivars, including those traditionally recalcitrant to transformation [15]. However, further work is necessary to eliminate the rhizogenic oncogenes, particularly the *rol* genes associated with hairy root formation. This requirement presents a significant limitation, as the approach is not readily applicable to species that do not primarily propagate through root proliferation.

## Floral Tissue Transformation

The floral dip transformation procedure, first pioneered in *Arabidopsis thaliana* by [12] and subsequently refined by [17], has significantly advanced plant genetic engineering. This technique involves dipping flowering inflorescences into an *Agrobacterium* suspension containing the desired gene construct. The *Agrobacterium* cells penetrate the ovules, delivering the transgene into the plant’s reproductive cells and resulting in the production of transgenic seeds. By enabling rapid and efficient generation of transgenic events, this method has transformed the genetic modification of *Arabidopsis*, solidifying its role as a model organism in plant research. Building on the success of floral dip, recent advancements have extended tissue culture-independent transformation methods to peanuts. The pollen tube transformation (PTT) method, coupled transformation efficiency in peanut, surpassing conventional tissue culture-based techniques. This method, which directly integrates target genes such as *AhBI-1* into the peanut genome through pollen tube injection, eliminates the need for complex tissue culture procedures, reducing cost and labor. Transgenic lines generated using this approach demonstrated enhanced root and shoot growth under normal conditions, underscoring the effectiveness of the technique for functional gene analysis and crop improvement. The simplicity, high efficiency, and tissue culture independence of PTT make it a valuable tool for advancing peanut genetic engineering (Fig. 4b) (Table 1) [87]. Recent studies have refined pollen tube injection protocols targeting the style cavity or keel petals within a two- to three-hour pre- and post-bloom window, achieving transformation frequencies averaging 2.6%. Basta screening emerged as a practical method for identifying heritable transgenic events, reducing the need for aseptic culture. Additionally, a DsRed fluorescence marker and flowering locus T (FT) overexpression enhanced flower abundance, facilitating transgenic progeny production. While efficiencies remain below earlier reports, these findings underscore the potential of pollen tube injection as a promising tissue culture-free method for peanut genetic transformation [28].

## High-Throughput Molecular Screening

An efficient and robust selection method is essential to enhance transformation efficiency and improve the quality of transgenic events. Organogenesis generally occurs when groups of cells develop into new shoot or root meristems during the regeneration process [46]. Effective selection is crucial to reduce chimerism in tissue culture-free transformation systems. Without sufficient selection pressure during shoot regeneration, both transgenic and non-transgenic meristematic cells proliferate, leading to regenerated shoots that are often chimeric. Since transgenic cells typically represent only a small fraction of the total meristematic population, inadequate selection results in a predominance of non-transgenic or mixed tissues, thereby necessitating the screening of numerous plants and diminishing the overall efficiency of recovering heritable transgenic events. Unlike conventional *in vitro* methods, the organogenesis in tissue culture-free transformation systems relies on the continuous growth of the mother explant, which supports the ongoing development of transgenic cells and rapid organ regeneration. Therefore, a careful balance, often achieved by applying sublethal herbicide levels is needed to selectively inhibit non-transgenic cell proliferation while promoting transgenic growth. For instance, wounded meristem tissue from germinated watermelon seeds inoculated with *Agrobacterium* and subsequently placed on a selection medium yielded a transformation efficiency of 17% [72]. A recent study [27] demonstrates a good alternative method through the use of the DsRed2 visual reporter in peanut genetic transformation. By enabling early and continuous visual detection of transgenic cells, from callus formation through to mature seed development, this approach increased the positive screening rate from 56.9 to 100%. Such findings highlight the potential of integrating robust visual markers into high-throughput transformation workflows, ultimately reducing the reliance on conventional molecular screening methods and improving overall throughput. Despite their simplicity and reliability, most of these protocols rely heavily on performing selection on solid media under sterile tissue culture conditions, which limits the overall throughput of the transformation process [3, 33]. By contrast, efficiency can be markedly improved by implementing selection on soil-grown plants, thereby broadening the applicability and throughput of transformation methodologies [23].

## Perspective

High-throughput transformation strategies in peanut focus on accelerating the development of transgenic or edited lines at scale while minimizing labor, time, and costs. One significant factor for achieving high-throughput transformation in peanut is the abundant availability of cost-effective target tissues, such as seeds or immature embryos. Seeds, in particular, are easily stored and can be processed en masse, making them a practical choice for large-scale experiments [86]. Several peanut transformation protocols have demonstrated that the use of seed-derived explants, such as germinating seeds or de-embryonated cotyledons, can significantly mitigate the complexities inherent in traditional callus-based systems by streamlining the regeneration process. This shift toward seed-focused approaches not only streamlines the transformation pipeline but also allows for rapid organogenesis and straightforward selection processes.

Equally important are strategies that optimize time and space usage. Methods such as the GiFT (genotype-independent fast transformation) system accelerate the regeneration of transgenic events, enabling selections in soil under herbicide spray within a matter of weeks [86]. This reduces the need for large greenhouse or tissue-culture facilities, cutting both operational costs and labor. A fundamental challenge across all crops is the limited flexibility of genotypes. Because only certain genotypes are amenable to transformation, the costs of generating transgenic or edited events remain high, and effective screening for desired phenotypes requires producing many events, which further strains the transformation capacity. Coupled with automation—such as mechanized explant preparation or liquid-based culturing in Temporary Immersion Systems—these advances promise shorter development cycles and increased throughput, ultimately facilitating quicker incorporation of desirable traits into peanut breeding programs.

To further improve transformation and editing efficiency, innovative strategies must be pursued that integrate advanced technologies with biological methodologies. One promising avenue is the incorporation of hyperspectral imaging within tissue culture environments. By merging robotics with artificial intelligence, specifically through AI-driven hyperspectral imaging or machine learning algorithms, researchers can accurately and rapidly identify healthy explants, thereby optimizing the selection process. Additionally, several complementary approaches can enhance both transformation and editing outcomes. These include: (1) developing tissue culture–free systems to streamline operations; (2) employing viral delivery mechanisms for single-guide RNAs (sgRNAs) and Cas nucleases to facilitate precise genome editing; (3) refining nanoparticle-based reagent delivery methods for improved cellular uptake; and (4) engineering superior strains of *Agrobacterium*, developing artificial chromosome technologies, and designing tunable or synthetic promoters for controlled gene expression. Advancements in robotics and AI are anticipated to revolutionize peanut tissue culture processes. Mechanized sampling and automated molecular screening techniques, such as polymerase chain reaction (PCR) or next-generation sequencing, will likely become standard practice. By leveraging machine learning, artificial intelligence, and robotics, researchers can efficiently identify high-quality transgenic or edited lines for seamless transfer to soil and greenhouse environments.

## Outlook

Tissue-culture-free transformation approaches are poised to become a cornerstone of future peanut breeding programs. Over the next few years, we anticipate a convergent platform in which (i) seed, node, or meristem-based *Agrobacterium* inoculation serves as a genotype-independent entry point, already delivering transformation efficiencies of ~ 30% in half-seed explants and above 50% via node injection; (ii) Virus-derived replicons or nanoparticle carriers can deliver multiplexed CRISPR-Cas genome editors. For example, a TYLCV-Cas12a replicon introduced via short-root vacuum infiltration induced targeted mutations in the peanut allergen genes *Ara h1, Ara h2, Ara h3,* and *Ara h6,* while pollen-magnetofection approaches are also being explored. These virus- and nanoparticle-mediated tools already let researchers validate gene targets rapidly through transient expression or somatic editing, providing a fast track to prioritize edits before committing to full breeding cycles. When integrated, the modules could, within a single ~ 120-day offseason, routinely yield transgene-free plants carrying stacked edits for reduced immunogenic proteins, fungal resistance, drought- and salinity-tolerance, elevated oleic-to-linoleic ratios, and increased oil content. Such capability would compress breeding timelines from multiple years into a one-cycle edit-and-fix process, fundamentally reshaping peanut-improvement pipelines. Beyond allergen-safe products for high-income markets, the same framework can expedite climate-resilient, oil-rich, high-yielding cultivars for smallholder farmers across semi-arid regions of Africa, South Asia, and Latin America, enhancing food security while safeguarding production against heat stress, salinity, and aflatoxin contamination. Continued refinement, higher editing efficiencies and more consistent inheritance should bring routine, on-demand trait introduction into elite cultivars across diverse peanut germplasm ever closer.

## Data Availability

All data are provided within the article itself.
